# Advances in Mesenchymal stem cells regulating macrophage polarization and treatment of sepsis-induced liver injury

**DOI:** 10.3389/fimmu.2023.1238972

**Published:** 2023-10-25

**Authors:** Yuhao Chen, Lihong Yang, Xihong Li

**Affiliations:** ^1^ Department of Emergency Medicine, West China Second Hospital of Sichuan University, Sichuan, China; ^2^ Key Laboratory of Birth Defects and Related Diseases of Women and Children, Sichuan University, Ministry of Education, Sichuan, China

**Keywords:** mesenchymal stem cells (MSCs), sepsis, liver injury, kupffer cells, macrophage polarization

## Abstract

Sepsis is a syndrome of dysregulated host response caused by infection, which leads to life-threatening organ dysfunction. It is a familiar reason of death in critically ill patients. Liver injury frequently occurs in septic patients, yet the development of targeted and effective treatment strategies remains a pressing challenge. Macrophages are essential parts of immunity system. M1 macrophages drive inflammation, whereas M2 macrophages possess anti-inflammatory properties and contribute to tissue repair processes. Mesenchymal stem cells (MSCs), known for their remarkable attributes including homing capabilities, immunomodulation, anti-inflammatory effects, and tissue regeneration potential, hold promise in enhancing the prognosis of sepsis-induced liver injury by harmonizing the delicate balance of M1/M2 macrophage polarization. This review discusses the mechanisms by which MSCs regulate macrophage polarization, alongside the signaling pathways involved, providing an idea for innovative directions in the treatment of sepsis-induced liver injury.

## 1 Introduction

### 1.1 Sepsis-induced liver injury

In 2016, the Sepsis-3 Workgroup introduced revised definitions for sepsis and septic shock, aiming to enhance accuracy and clinical identification. Sepsis is now defined as a critical condition wherein organ dysfunction arises from a dysregulated host response to infection. Septic shock is identified by the clinical requirement of vasopressors to maintain a mean arterial pressure equal to or above 65 mmHg, accompanied by a serum lactate level exceeding 2 mmol/L, without evidence of hypovolemia (1). Liver injury is a familiar organ damaged in patients with sepsis. It can be viewed as a primary dysfunction that occurs within the first hour after the initial injury, which is usually associated with liver hypoperfusion. This can result in diffuse intravascular coagulation and multiple organ failure (2). Concurrently, sepsis can induce dysfunction in the intestinal microcirculation, facilitating the infiltration of intestinal toxins and bacteria into the liver through the portal vein, thereby initiating a cascade of hepatic inflammation. Within the context of sepsis, the liver becomes a hub of intensified oxidative stress reactions, generating byproducts that activate neutrophils and exacerbate hepatic damage (3). There is evidence that liver injury and failure, particularly as a severe complication of sepsis, contribute directly to disease progression and patients’ death (4).

Sepsis is most common in people with weakened immune systems, such as the elderly, infants and patients with certain underlying medical conditions. Sepsis will become more common as the number of elderly patients grows (5). The average annual increase percentage in sepsis incidence was 13%-13.3% (6). Currently, the main way to improve outcomes is to recognize sepsis early and treat it appropriately in the initial hours. However, targeted and effective treatment strategies are still lacking. As a result, it is urgent to investigate a treatment for sepsis-induced liver injury.

### 1.2 Macrophage

**Figure 1:**
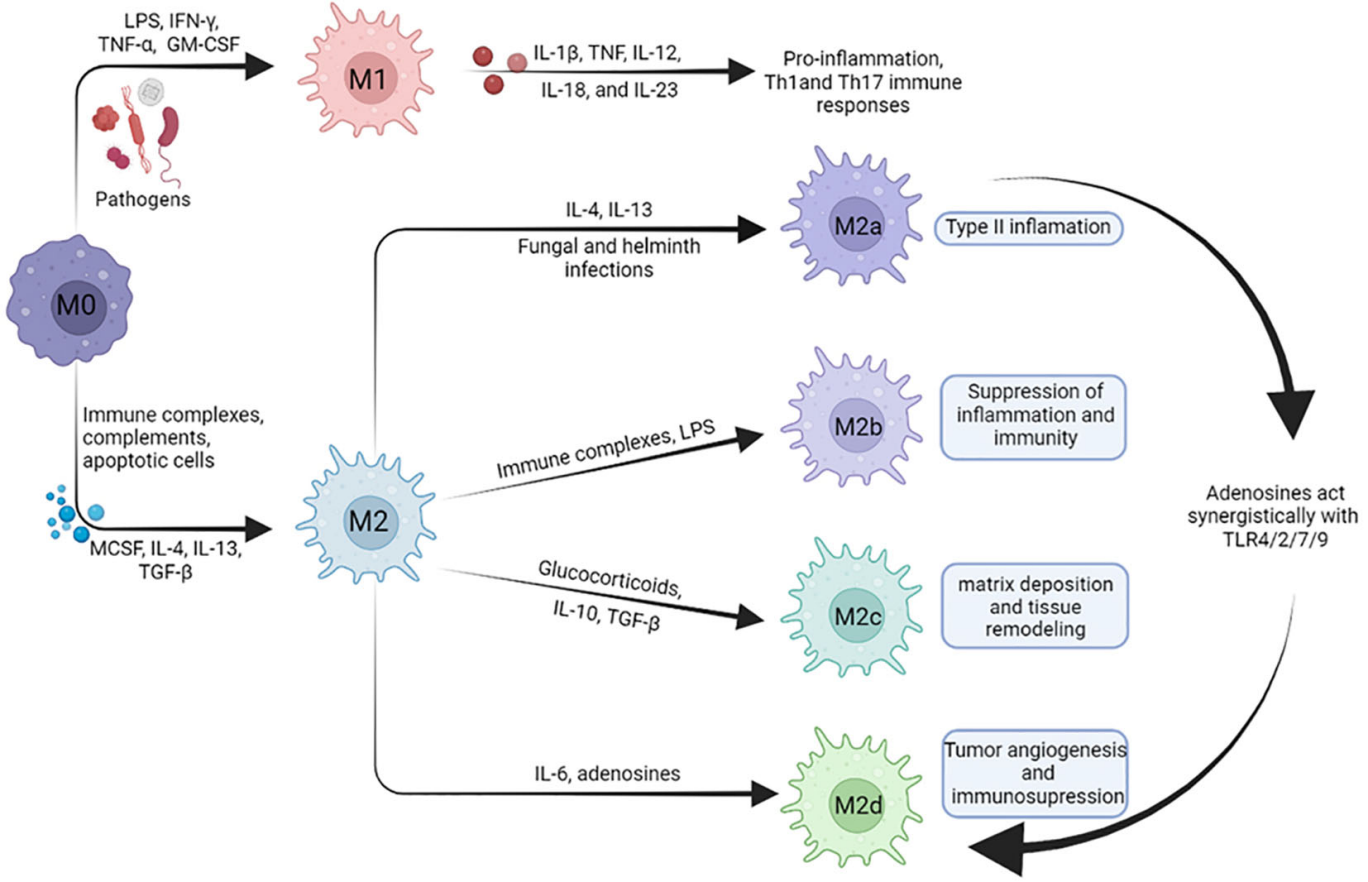
Macrophages exhibit remarkable plasticity and can undergo polarization into either M1 macrophages or M2 macrophages in response to specific microenvironmental cues. M1 macrophages are characterized by the secretion of various pro-inflammatory cytokines and inflammatory mediators, contributing to tissue damage and robust inflammatory responses. M2 macrophages has been subdivided into distinct subtypes, including M2a, M2b, M2c, and M2d. Under certain conditions, M2a can be transformed into M2d. Activated M2a macrophages can be involved in type II inflammation. M2b macrophages can be involved in the supression of inflammation and immunity response. M2c macrophages can be involved in matrix deposition and tissue remodeling. M2d macrophages can be involved in tumor angiogenesis and immunosupression.

Kupffer cells, the specialized macrophages residing in the liver, distinguish themselves from monocyte-derived macrophages by their distinct localization and rapid accumulation within the damaged liver. As resident tissue macrophages, Kupffer cells exhibit a mature phenotype that demonstrates remarkable plasticity. Their functional activity dynamically evolves in response to the specific metabolic and local immune environment (7). Macrophages exhibit a classification into two distinct phenotypes: M1 and M2. M1 macrophages, also known as classically activated macrophages, possess the ability to release elevated levels of proinflammatory cytokines, including TNF-α, IL-6, IL-12, and inducible nitric oxide synthase (iNOS). In contrast, M2 macrophages, alternatively activated macrophages, display a different profile. They tend to release lower levels of proinflammatory cytokines and instead exhibit higher levels of anti-inflammatory mediators, such as IL-10 and transforming growth factor beta (TGF-β) (8). Therefore, inhibition of excess polarization of M1 macrophages and promotion of polarization of M2 macrophages in patients with sepsis may help improve the condition (9). Emerging data has revealed significant heterogeneity even within the traditional M1 and M2 macrophage classifications, underscoring the oversimplification of the M1/M2 categorization. In fact, the M2 phenotype has been further subdivided into distinct subtypes, namely M2a, M2b, M2c, and M2d, reflecting the diverse functional states and responses exhibited by these macrophages (**Figure 1**). The subtypes have unique cell surface marker proteins and distinct functions, and they are induced by various regulators (10). This refined categorization highlights the intricate and multifaceted nature of macrophage polarization, emphasizing the need for a more comprehensive understanding of the various subpopulations and their specific roles in immune regulation, inflammation, and tissue repair.

### 1.3 The role of macrophages in sepsis

#### 1.3.1 Bacterial clearance

The liver is an essential component of the inflammatory response and is crucial for germ limitation and toxin elimination in sepsis (11). In animal models, more than 60% of the total number of bacteria can be eliminated from the blood and limited in the liver after 10 minutes, and more than 80% of them can be limited in the liver after 6 hours (12). In bacterial infections, lipopolysaccharide (LPS) serves as a crucial inflammatory trigger. Notably, the liver plays a pivotal role in eliminating LPS from the circulation (13). When the liver sustains damage, its capacity for efficient bacterial clearance becomes compromised. This impairment in bacterial clearance increases the risk of sepsis and systemic infection can spread unchecked. Thus, liver damage becomes a significant factor contributing to the heightened vulnerability to sepsis (14). The mediation of bacterial phagocytosis and clearance within the liver involves a diverse range of cells. These cells operate as the initial line of defense against the translocation of bacteria through the bloodstream. The active participation in this process is undertaken by Kupffer cells, liver sinusoidal endothelial cells (LSECs), and stellate cells (15). Kupffer cells, as resident macrophages inhabiting the hepatic sinusoids, are cells with an exceptional capacity for phagocytosis. These specialized cells play a crucial role in the liver’s immune defense system by efficiently removing bacteria and soluble bacterial products from the bloodstream (2, 16).

Platelets and neutrophils work along with Kupffer cells to remove bacteria from the blood. Platelets release many antimicrobial molecules and play a direct role in infection defense. Platelets can also enhance the killing capacity of Kupffer cells (16). By secreting Kupffer cell chemokines, neutrophils move and gather in the hepatic sinusoids during sepsis. Then, neutrophils and platelets interact to jointly promote the release of neutrophil extracellular traps to trap and clear pathogens (17). The impaired bacterial clearance observed in the liver during sepsis is attributed to a combination of factors, including the direct impact of reduced platelet count on immune responses, the damage inflicted on the reticuloendothelial system responsible for bacteria phagocytosis and clearance, and the compromised function of neutrophils, leading to reduced phagocytosis and intracellular killing capacity (18).

#### 1.3.2 Liver-mediated pro-inflammatory response

In sepsis patients, the liver serves as a prominent site of inflammatory responses triggered by bacterial endotoxins. Additionally, the liver itself can contribute to the production and release of inflammatory mediators. Meanwhile, other organs may have significant and deadly inflammatory reactions because of the damaged liver (19).

Kupffer cells are in charge of generating inflammatory cytokines and mediating liver injury in the early stages of sepsis. Upon encountering harmful bacteria or endotoxins, Kupffer cells respond by augmenting the release of several early proinflammatory mediators. These include IL-1, IL-6, IL-8, TNF-α, IFN-γ, and monocyte chemotactic protein 1 (11). Studies have shown that inducing Kupffer cell exhaustion through the administration of gadolinium chloride before cecal ligation and puncture (CLP) can have beneficial effects during the early stages of sepsis in rats. This exhaustion of Kupffer cells leads to a reduction in the secretion of pro-inflammatory cytokines. Additionally, it helps improve hepatic microcirculation disorders, reduce hepatic cell apoptosis, and prevent the development of liver injury. However, hepatic bacterial clearance impaired due to Kupffer cells loss, finally the survival of septic rats was remarkably decreased (20). The modulation of Kupffer cell differentiation represents a novel strategy with the potential to suppress inflammation and protect organs from injury.

Inflammation and chemokine production are also mediated by hepatocytes, hepatic stellate cells, and LSECs. Pathogen proteins are identified by hepatic stellate cells and LSECs through pattern recognition receptors such as the Toll-like receptor (TLR), enabling them to assume the role of liver antigen-presenting cells. In collaboration with Kupffer cells, these cells orchestrate a series of immunological events in sepsis. They activate hepatic natural killer T cells, classical T cells (CD4+ and CD8+ T lymphocytes), recruit neutrophils to the liver, and initiate both local and systemic inflammatory responses (2).

#### 1.3.3 Liver-mediated immunosuppression

Liver has a special innate immune microenvironment and plays a crucial part in surveillance for immune homeostasis (21, 22). Due to its unique dual blood supply, the liver is subjected to a constant exposure to circulating antigens, pathogens, and pathogen-associated toxins. These agents gain access to the liver through multiple routes, including the gastrointestinal tract, portal vein, and systemic circulation via arterial blood (23). Thus, liver cells act as gatekeepers to initiate or suppress immune responses as needed. The liver harbors a significant population of intra-tissue macrophages, primarily represented by Kupffer cells located within the hepatic sinusoids. These Kupffer cells serve as the predominant phagocytic cells in the liver and constitute more than 80% of the macrophage population in a healthy human liver. Additionally, it contains lymphoid (such as natural killer cells, T cells, or B cells) and myeloid (such as neutrophils or macrophages) immune cells, which collectively compose both innate and acquired immune responses (22).

The complexity of sepsis ranges from the initial stage of inflammatory response to the later stages of immunosuppression (24). The initial phase manifests as systemic inflammatory response syndrome, which include systemic inflammation, cytokine storm and multi-organ damage (25). When the body’s immune cells detect bacteria or endotoxins, a potent inflammatory response is triggered. If the process is not properly controlled, monocytes become unable to respond to further endotoxin attacks, and they begin to produce anti-inflammatory cytokines (TGF-β, IL-10), the spesis will enter a state of immunosuppression. Endotoxin tolerance is one of the main mechanisms of immunosuppression in sepsis. It is the diminished reactivity to endotoxin challenge following the initial exposure (26, 27). Endotoxin tolerance can protect body from lethal endotoxin attack and prevents infection and ischemia-reperfusion injury. In the meantime, endotoxin tolerance has a significant impact on patients’ vulnerability to reinfection, which in septic patients can be fatal (24). Uncontrolled inflammatory responses produce cytokine storms that lead to abroad tissue damage and pathological manifestations states such as sepsis (27).

### 1.4 Mesenchymal stem cells

Mesenchymal stem cells (MSCs) possess an immense capacity for self-renewal and multi-differentiation. These remarkable cells can be sourced from various tissues, such as bone marrow, adipose tissue, umbilical cord, and placental tissue (28, 29). MSCs exhibit a variety of advantageous characteristics in inflammatory diseases, including the ability of homing, reduce inflammatory response, regulate immune homeostasis, mitigate organ damage, and stimulate tissue regeneration (30). Based on the characteristics, MSCs have widespread application in the fields of cell therapies and bioengineering (28, 29). As a result, MSCs appear to be promising as one of the treatments for sepsis (31).

## 2 How MSCs regulate macrophage polarization

### 2.1 Paracrine effects of MSCs

**Figure 2:**
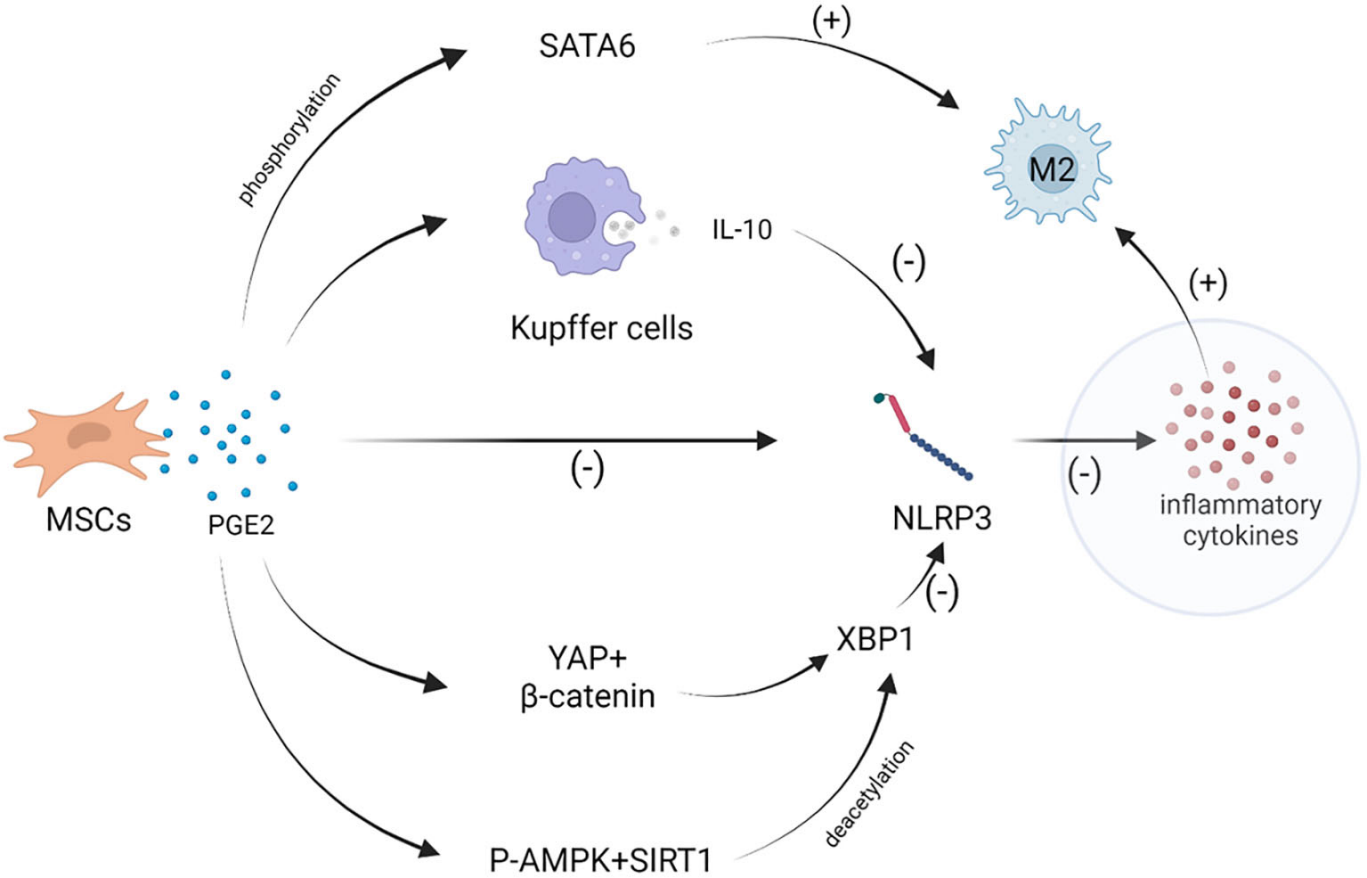
MSCs can paracrine PGE2 and affect the macrophages polarization through a variety of ways. PGE2 inhibits NLRP3 inflammasome to lessen the secretion of inflammatory cytokines. PGE2 control the Hippo-YAP pathway of macrophages and increase the expression of p-AMPK and SIRT1, then regulates XBP1 to reduce NLRP3 inflammasome activity, which promotes macrophages polarization toward M2 phenotype. In addition, PGE2 can stimulate the phosphorylation and activation of STAT6, induces macrophages to M2 polarization.

MSCs can reduce inflammatory response, promote tissue repair and regeneration through paracrine soluble factors (32). MSCs possess the capability to modulate the polarization of macrophages and regulate the secretion of inflammatory factors. Through the paracrine signaling of prostaglandin E2 (PGE2). PGE2 exerts inhibitory effects on the nucleotide-binding and oligomerization domain-like receptor 3 (NLRP3) inflammasome, resulting in the reduction of inflammatory cytokine secretion, including IL-1β, IL-6, and IL-18. By attenuating the activation of the NLRP3 inflammasome, MSCs can effectively mitigate acute liver inflammation, thereby contributing to the amelioration of liver injury and the restoration of immune homeostasis (33). MSCs control the Hippo-YAP pathway of macrophages in a mouse model of inflammatory liver injury by secreting PGE2 to prevent the phosphorylation of mammalian Ste20-like kinase 1/2 and large tumor suppressor 1, to boost the translocation of YAP from cytoplasm to nucleus. By directly interacting with YAP and β-catenin to activate NLRP3, the Hippo pathway then regulates XBP1 to reduce NLRP3/caspase-1 activity and IL-1 production, which in turn facilitate hepatic macrophages to polarize toward M2 phenotype (34). MSCs produce PGE2, which not only inhibits inflammatory cytokine secretion but also facilitates the polarization of hepatic macrophages towards the M2 phenotype (35, 36). This process is mediated by the activation and phosphorylation of the transcription factor signal transducers and activators of transcription 6 (STAT6). PGE2, through its interaction with specific receptors on macrophages, triggers the activation and phosphorylation of STAT6, a classical mechanism involved in M2 macrophage polarization. This shift towards the M2 phenotype promotes an anti-inflammatory environment and supports tissue repair processes in the liver, further contributing to the resolution of inflammation and the promotion of liver recovery (33, 35) (**Figure 2**).

**Figure 3:**
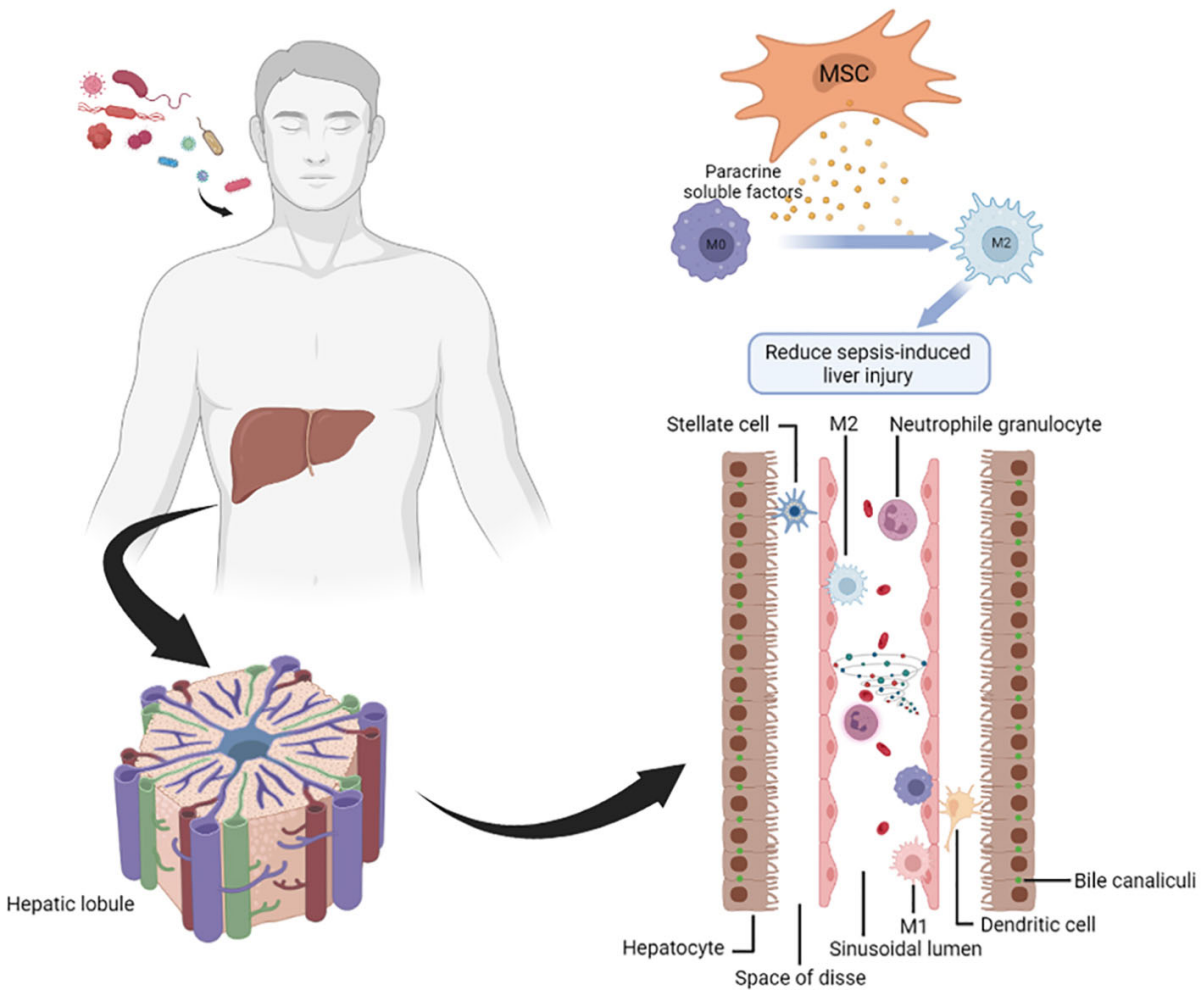
MSCs attenuate sepsis-induced liver injury through paracrine soluble factors. Immune cells, such as neutrophils and Kupffer cells, accumulates in the hepatic sinuses and produce a large number of cytokines, resulting in a cytokine storm. MSCs can play a pivotal role in attenuating sepsis-induced liver injury through the immunoregulation effects of paracrine soluble factor.

When the expression of MSCs-derived TSG-6 (TNF-α-stimulated gene 6) was inhibited, there was a notable impact on the macrophage population in the pancreatic and liver tissues of rats with severe acute pancreatitis. Specifically, the presence of iNOS+ M1 macrophages significantly increased, while the abundance of CD163+ M2 macrophages significantly decreased. Inhibition of TSG-6 expression disrupts this regulatory mechanism, leading to an imbalance in macrophage phenotypes and potentially exacerbating the inflammatory state associated with severe acute pancreatitis in the pancreatic and liver tissues of rats (37). Within the inflammatory state of the host, the generation and secretion of IL-4 by bone marrow-derived MSCs occur, triggering the activation of host liver macrophage reprogramming. Simultaneously, the upregulation of Wnt-3a expression is induced by MSCs. Facilitating the shift from the M1 pro-inflammatory phenotype to the M2 anti-inflammatory phenotype (38). Through the co-encapsulation of hepatocytes and human umbilical cord MSCs (HNF4α-UMSCs), a series of beneficial effects have been observed. This co-encapsulation approach has demonstrated the capacity to diminish liver damage induced by LPS/D-galactosamine, elevate the survival rate of mice with acute liver failure (ALF), and enhance the survival, proliferation, and metabolic function of hepatocytes. These positive outcomes are achieved by facilitating the polarization shift of macrophages from the M1 to the M2 phenotype, and leveraging paracrine mechanisms (39). Through the augmentation of HNF4α expression, an elevation in the transcriptional activity of IL-10 is achieved, consequently promoting the polarization of M2 macrophages via the activation of the IL-10/STAT3 pathway (40). Using paracrine soluble substances, especially through controlling macrophage activity, can help to reduce excessive inflammatory reactions (**Figure 3**).

### 2.2 Exosomes of MSCs

The International Society for Extracellular Vesicles (ISEV) endorses the term “extracellular vesicle” (EV) as the universal terminology for naturally released particles originating from cells. These vesicles are characterized by a lipid bilayer membrane, lack the ability to replicate and do not possess a functional nucleus within their structure (41). EVs are commonly categorized into three subtypes based on their size and biogenesis: exosomes (Exos), microvesicles, and apoptotic bodies. Exos are generated through the fusion of multivesicular bodies with the plasma membrane, resulting in their release into the extracellular space (42). EVs can carry complex macromolecular substances such as proteins and nucleic acids, and introduced into recipient cells to take a variety of biological effects (8). Exos serve as both providers of biologically active molecules and essential carriers to protect the molecules and deliver them to the appropriate targets. Exos are preferentially endocytosed in the injured tissue because Exos uptake is reliant on the acidity of the intracellular and microenvironment and tissue injury is typically characterized by acidosis (43).

The immunomodulatory effects displayed by MSC-Exos, akin to those exerted by MSCs, have been demonstrated both *in vitro* and *in vivo* settings (44, 45). MSC-Exos were found to repair injured liver tissue in ALF model mice and reduce the expression of NLRP3 inflammasome and caspase-1, IL-1β and IL-6 in acute liver failure (46), thereby promoting macrophage polarization toward the M2 phenotype (34). MSC-Exos accumulated in the liver 6 hours after injection in the mouse model of partial hepatectomy and was primarily absorbed by liver macrophages, MSC-Exos exert their hepatoregenerative effects through the modulation of macrophage phenotypic transformations (45). Anti-inflammatory-related miRNA-299-3p had been found up-regulated in TNF-α pretreatment of umbilical cord MSC-derived Exos. Their high expression may contribute to the reduction of blood levels of alanine aminotransferase (ALT), aspartate aminotransferase (AST), pro-IL-1 and pro-IL-18, pro-inflammatory cytokines, attenuation of liver injury, and inhibition of NLRP3 inflammation-associated pathway proteins (47). The miRNA-17, abundant in MSC-Exo cargo, can also suppress NLRP3 inflammasome activation by targeting thioredoxin-interacting protein (TXNIP) in macrophages (48). The miRNA-182-5p was significantly enriched in MSC-Exos. By preventing the production of the forkhead box transcription factor 1 (FOXO1) in macrophages, the miRNA-182-5p of MSC-Exos reduced the expression of the TLR4 and triggered an anti-inflammatory response (45).

Experiments conducted *in vitro* have demonstrated that MSC-Exos decrease inflammatory responses and may control macrophage polarization by preventing hypoxia-inducible factor 1 (HIF-1) from mediating glycolysis, significantly inhibiting M1 polarization and promoting M2 polarization (49). It was discovered by Zhang Y et al. that Kupffer cell M2 polarization is dependent on the presence of IL-10 within MSC-EVs, as opposed to free IL-10. The EVs carrying IL-10 were collected by Kupffer cells, subsequently inducing the expression of PTPN22. This, in turn, facilitated macrophage polarization towards the M2 phenotype, leading to a reduction in liver inflammation and damage (50).

In the study conducted by Siyuan et al., it was demonstrated that miRNA-148a, which is enriched within the extracellular vesicles (Exos), exerts regulatory effects on Kruppel-like factor 6 (KLF6). Through this regulatory interaction, miRNA-148a exhibits the capability to suppress M1 macrophages while simultaneously promoting the polarization of M2 macrophages. This modulation is achieved by inhibiting the STAT3 pathway (51). However, Hui et al. found that MSC-Exos induced macrophage polarization toward M2 with arginase-1 high expression mainly through transporting the activated STAT3 (52). The mechanism of regulating STAT3 pathway affecting macrophage polarization needs to be further studied.

### 2.3 Homing

Stem cell homing refers to the process in which autologous or exogenous stem cells can migrate to target tissues and colonize under the action of various factors (53). In a mouse model of sepsis-induced liver injury, the use of iron oxide-based synthetic nanoparticles containing MSCs (SPION-MSCs) was found to facilitate the polarization of macrophages towards the M2 phenotype. The introduction of SPIONs did not compromise the fundamental characteristics of MSCs. Instead, it stimulated the expression of Haem oxygenase 1 within MSCs, allowing for the regulation of their activity within an inflammatory environment (9). Following infusion, SPION-MSCs exhibited a rapid homing to the lungs and subsequently became trapped in the liver for a period exceeding 10 days. In contrast, their residence in other organs was infrequently observed. Importantly, the promotion of M2 macrophage polarization was attributed to the phagocytosis of SPION-MSCs by these macrophages. This phenomenon suggests that the interaction between SPION-MSCs and M2 macrophages plays a significant role in facilitating the polarization of macrophages towards the M2 phenotype (54). Additionally, the expression of TNF receptor-associated factor 1 by SPION-MSCs was found to be crucial for the promotion of macrophage polarization and the subsequent reduction of sepsis in mice (9).

In conclusion, the regulation of macrophage polarization by MSCs can occur through various mechanisms, including the secretion of paracrine soluble factors, the release of Exos, and the process of homing (**Table 1**). Consequently, this regulatory capacity holds great promise as a therapeutic approach for addressing sepsis-induced liver injury.

**Table 1:** The ways of MSCs regulate macrophage polarization.

Authors	Publication time	Sources of MSCs	Regulation of macrophage polarization	Results
Lee KC et al. (55)	2015	Mice bone marrow	Paracrine soluble factors: IL1Ra	MSCs reduced liver injury by increasing IL10 production through an IL1Ra dependent M2 polarization of macrophage.
Miao CM et al. (36)	2016	Mice bone marrow	Paracrine soluble factors: PGE2	MSCs secrete PGE2 to inhibit M1 Kupffer cells and promote M2 Kupffer cells, enhancing IL-10 release.
Liu Y et al. (48)	2018	Mice adipose	MSCs-derived exosomal miRNA -17	MSC-Exos protect against ALF by inhibiting NLRP3 inflammasome activation in macrophages via miR-17-mediated TXNIP inhibition.
Li C et al. (34)	2019	Mice bone marrow	Paracrine soluble factors: PGE2	Mesenchymal stem cells promoted the macrophage Hippo pathway, reduced NLRP3/caspase-1 activity, leading to reprogramming of macrophages to the M2 phenotype.
Jiang L et al. (46)	2019	Human umbilical cord	MSC-Exos	MSC-Exos repaired liver tissue, reducing NLRP3 inflammasome expression and levels of ALT and AST in a mouse ALF model.
Kong D et al. (39)	2020	Human umbilical cord	Paracrine soluble factors:HB-EGF	Co-encapsulation of HNF4α-MSCs and hepatocytes promoted M2 macrophage polarization and attenuated the inflammatory response.
Zhang S et al. (47)	2020	Human umbilical cord	MSCs-derived exosomal miRNA-299-3p	T-Exos effectively reduced serum levels of ALT, AST, and proinflammatory cytokines, suppressed the activation of NLRP3 inflammation-associated pathway proteins, and mitigated pathological liver damage in ALF.
Wang J et al. (33)	2021	Mice bone marrow	Paracrine soluble factors: PGE2	MSC-derived PGE2 induces M2 macrophage polarization in liver through STAT6 and mTOR signaling pathways.
Chang CY et al. (52)	2021	Human placenta choriodecidual membrane	MSCs-derived exosomal let-7i-5p miRNA	Exos therapy restores metabolic homeostasis, reduces oxidation and inflammation, and promotes the polarization of macrophages towards the M2 phenotype.
Zhang Y et al. (50)	2021	Mice bone marrow	MSCs-derived EVs containing IL-10	IL-10-containing EVs were captured by Kupffer cells, leading to the upregulation of protein tyrosine phosphatase non-receptor 22 (PTPN22). This resulted in the transformation of Kupffer cells into the M2 phenotype, effectively mitigating liver inflammation.
Xu Y et al. (9)	2021	Human umbilical cord	Homing of MSCs	The enhanced macrophages polarization towards the M2 phenotype observed in sepsis-induced liver injury in mice is attributed to the phagocytosis of SPION-MSCs.
Sun W et al. (44)	2022	Human umbilical cord	MSC-Exos	Exos derived from MSCs exhibit potent anti-inflammatory and immunosuppressive effects, thereby preventing critical organ injury by promoting the polarization of M2 macrophages and regulatory T cells.
Tian S et al. (51)	2022	Human umbilical cord	MSCs-derived exosomal miRNA-148a	MiR-148a, abundant in MSC-Exos, selectively targets Kruppel-like factor 6 (KLF6) to suppress M1 macrophages while promoting M2 macrophages through the inhibition of the STAT3 pathway.
Wang J et al. (38)	2022	Mice bone marrow	MSCs-derived exosomal IL-4	Transplanting allogeneic MSCs alleviated ALF by inducing a switch of macrophages towards the M2 phenotype, and this effect was dependent on IL-4.
Xu J et al. (45)	2022	Mice bone marrow	MSCs-derived exosomal miRNA- 182- 5p	Hypoxic MSC-Exos promote macrophage polarization to the M2 phenotype during liver regeneration by modulating the FOXO1/TLR4 signaling pathway.
Yu Y et al. (40)	2022	Human umbilical cord	Paracrine soluble factors: IL-10	Overexpressing HNF4α in MSCs alleviated ALF and induced macrophage polarization to the M2 phenotype via the IL10/STAT3 pathway.
Watanabe Y et al. (56)	2022	Human gingival tissue	MSCs-derived exosomal CD73, CD5L	MSC-Exos, when stimulated with the combination of TNF-α and IFN-α, enhance macrophage polarization to the M2 phenotype through the upregulation of exosomal CD73 and CD5L.

## 3 MSCs-regulated signaling pathways of macrophage polarization

### 3.1 NF-κB signaling pathway

NF-κB is a universal transcription factor and a critical regulator of gene expression during severe infections, including sepsis (57). It is one of important transcription factor associated with the activation of M1 macrophages (58). Studies demonstrated that inhibition of NF-κB activation by MSCs can remarkably reduce sepsis-induced liver injury (59). Therefore, inhibition of NF-κB pathway by MSCs may be a significant molecular mechanism in the treatment of sepsis-induced liver injury. P50 NF-κB protein can inhibit NF-κB signaling pathway, and activate the M2 polarization (60). The miRNA-27b supplied by MSC-derived exos could decrease the inflammatory response and prevent sepsis by downregulating p65 NF-κB, which can activate the NF-κB signal pathway (61). Jie et al. found that small EVs from MSCs can limit the phosphorylation of the NF-κB pathway (62). Thus, EVs acting on the NF-κB pathway may be one of the effective ways to treat sepsis.

### 3.2 JAK/STAT signaling pathway

The Janus family of kinases (JAK) encompasses four major members, namely JAK1, JAK2, JAK3, and Tyk2. These proteins, belonging to the tyrosine kinase family, exhibit high homology and share similar structural characteristics (63). Many cellular functions are reliant upon the pivotal role played by the STAT (Signal Transducer and Activator of Transcription) family, consisting of essential members such as STAT1, STAT2, STAT3, STAT4, STAT5A, STAT5B, and STAT6 (64). To regulate the expression of associated genes, the JAK enzymes are capable of phosphorylating STAT proteins, giving rise to what is commonly referred to as the JAK-STAT signaling pathway. This intricate pathway exerts significant control over immunological responses, cell growth, proliferation, and differentiation processes (63).

An investigation found that the potential functions of the JAK/STAT pathway in regulating the systemic inflammatory response elicited by septic challenge were examined *in vivo*. The researchers observed that JAK2 exhibited rapid activation in septic rats, with maximal activation occurring in hepatic tissues after 6 hours. Notably, in septic rats induced by CLP, they demonstrated that the JAK/STAT pathway could potentially exert control over the development of organ damage in various organs. These findings shed light on the role of the JAK/STAT pathway in the pathogenesis of sepsis and suggest its involvement in orchestrating the inflammatory response and subsequent organ injury during septic conditions (65). A study found that inhibiting the JAK2/STAT3 signaling pathway might diminish the production of proinflammatory cytokines including TNF-α and IL-6, as well as mitigate multiple organ failure in severe sepsis (66). Lentsch et al. made an intriguing discovery regarding the dysregulated activation of the transcription factor NF-κB in STAT6-deficient mice. This dysregulation led to an augmented production of pro-inflammatory cytokines and chemokines in the liver, including MIP-1, MIP-2, IP-10, and MCP-1. Additionally, upon endotoxin stimulation, STAT6-deficient animals exhibited a higher accumulation of neutrophils and leukocytes within the liver. This enhanced accumulation of immune cells may potentially contribute to organ damage (67).

Mesenchymal stem cells stimulate the phosphorylation and activation of STAT6 by paracrine PGE2, which in turn induces macrophages to M2 polarization. Increasing M2 macrophages by MSC treatment can activate the IL-4/STAT6 signaling pathway to control the acute-phase response in the liver (35).

### 3.3 AMPK/SIRT1 signaling pathway

AMP-activated protein kinase (AMPK) is an important regulator of energy metabolism at the cellular level. Sirtuin (SIRT) is a NAD^+^-dependent protein deacetylase, SIRT1 is one of the most concerned members, it plays a key role in the regulation of inflammation, immune response, metabolism and apoptosis/aging. In the aspect of maintaining energy homeostasis, AMPK and SIRT1 often show synergistic effect, and also interact to regulate each other’s expression. The target of AMPK/SIRT1 is a classical upstream signaling pathway of oxidative stress and crucial for maintaining metabolic homeostasis (68). Jagged1 treatment significantly raised the amount of PGE2 that MSCs secreted. PGE2 then increased the expression of p-AMPK and SIRT1, which in turn caused XBP1s to be deacetylated and the NLRP3 inflammasome to be inhibited in macrophages (69), thereby promoting macrophage polarization toward the M2 phenotype (34).

### 3.4 Notch signaling pathway

Recent studies have highlighted the participation of the Notch pathway in critical processes such as liver regeneration and repair, liver fibrosis, and metabolism. These findings suggest that the Notch signaling pathway plays a significant role in maintaining liver homeostasis and responding to physiological and pathological changes within the liver (70). Notch signaling pathway is crucial in macrophage polarization (71). It can up-regulate miRNA-148a-3p expression in macrophages, when miRNA-148a-3p can accelerate M1 polarization of macrophages (72). Through activation of NF-κB, activated Notch1 and expression of the Notch target genes remarkably regulate the production of TNF-α, IL-6, and IL-10 (71). MSC transplantation remarkably reduced Notch1 receptor in liver failure rats, suppressing the M1 polarization of macrophage. The impact of MSCs on hepatocyte regeneration may be influenced by the down-regulation of Notch signaling (73). Further investigations into the intricate mechanisms underlying Notch pathway regulation hold promise for developing novel therapeutic strategies targeting liver-related disorders.

## 4 MSCs treatment sepsis-induced liver injury

As the liver serves as the primary defense against infections and also plays a crucial role in drug metabolism, it is susceptible to injuries induced by both infections and drugs. In a study conducted, mice were intravenously administered with MSCs one hour before being subjected to a CLP challenge. Following the CLP challenge, there was a significant increase in the levels of AST and ALT. However, the intervention involving the administration of MSCs effectively mitigated the elevated levels of AST and ALT, alleviated pathological injury of the liver and enhanced the survival rate of mice in the sepsis model (74).

When MSCs were administered into a mouse model with CLP-induced sepsis, there was a notable attenuation in the expression of TNF-α and IL-6, while concurrently witnessing an upsurge in the expression of IL-4 and IL-10. This not only mitigated the pronounced hepatic swelling and necrosis observed in the liver but also led to a decline in the elevated levels of AST and ALT. Additionally, there was a discernible reduction in the presence of Bax- and Caspase-3-positive apoptosis cells, coupled with an enhanced glycogen deposition within the liver, ultimately contributing to an improved survival rate (59, 75). It’s noteworthy to mention that SPION-MSCs exhibited a more pronounced ameliorative effect on these pathological symptoms in both CLP and LPS sepsis mouse models in comparison to when MSCs were used in isolation (9).

## 5 Conclusion

In summary, the modulation of macrophage polarization by MSCs offers a promising therapeutic approach for sepsis-induced liver injury. The paracrine secretion of soluble factors, exosomes, and the ability of MSCs to home to the liver contribute to their beneficial effects in reducing liver injury and promoting tissue repair. Further understanding of the signaling pathways involved and optimization of MSC-based therapies will pave the way for their clinical application in treating sepsis-induced liver injury, offering new hope for patients facing this challenging condition.

## Author contributions

YC: Writing - Original draft. LY and XL: Writing -Review and Editing. All authors contributed to the article and approved the submitted version.
